# Supplementary material for: Morphology, Micromechanical, and Macromechanical Properties of Novel Waterborne Poly(urethane-urea)/Silica Nanocomposites
Source: Materials (Basel). 2023 Feb 21;16(5):1767. doi: 10.3390/ma16051767 (PMC10004705; doi:10.3390/ma16051767)
Supplement: Supplementary file 1 [file materials-16-01767-s001.zip › materials-2141449-supplementary.pdf]

SUPPLEMENTARY MATERIALS

Morphology, Micromechanical, and Macromechanical Properties of Novel Waterborne Poly(urethane-urea)/Silica Nanocomposites

Veronika Gajdošová, Milena Špírková, Yareni Aguilar Costumbre, Sabina Krejčíková, Beata Strachota, Miroslav Šlouf * and Adam Strachota *

Institute of Macromolecular Chemistry, Czech Academy of Sciences, Heyrovskeho nam. 2, CZ-162 00 Praha, Czech Republic

* Correspondence: slouf@imc.cas.cz (M.Š.); strachota@imc.cas.cz (A.S.)

1 Synthesis

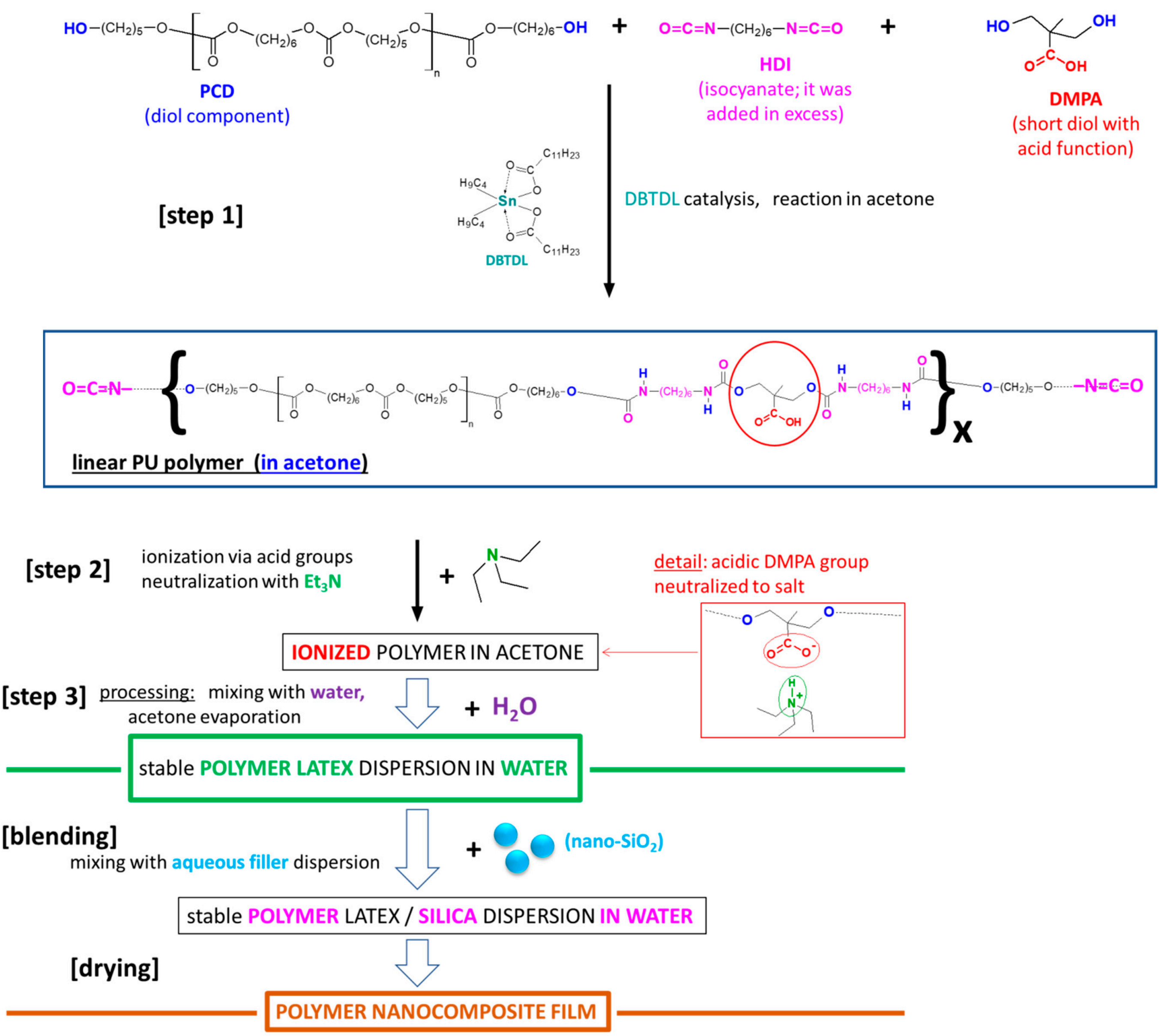

Scheme S1. *Synthesis of the studied polyurethane nanocomposites.*

2 Segmented structure of the PUU matrix

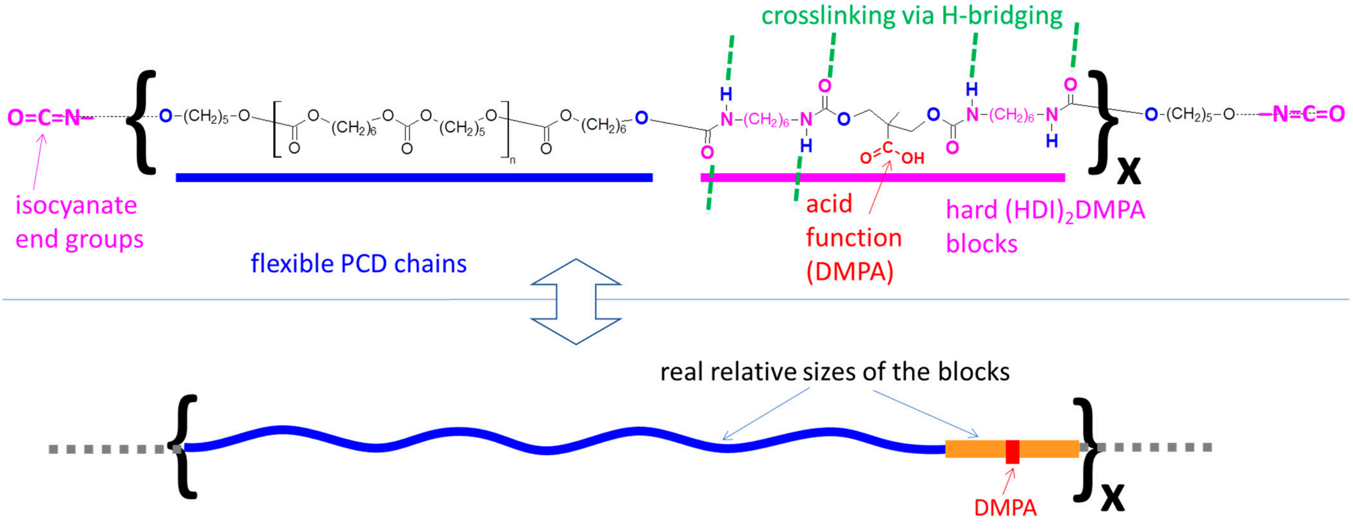

Scheme S2. *(Top) idealized chemical structure and (bottom) symbolic representation of the polymer matrix of the studied nanocomposites, which contains relatively inert flexible ‘soft segments’, as well as strongly and multiply hydrogen-bonding ‘hard segments’.*

3 Morphology: TEM/SEM

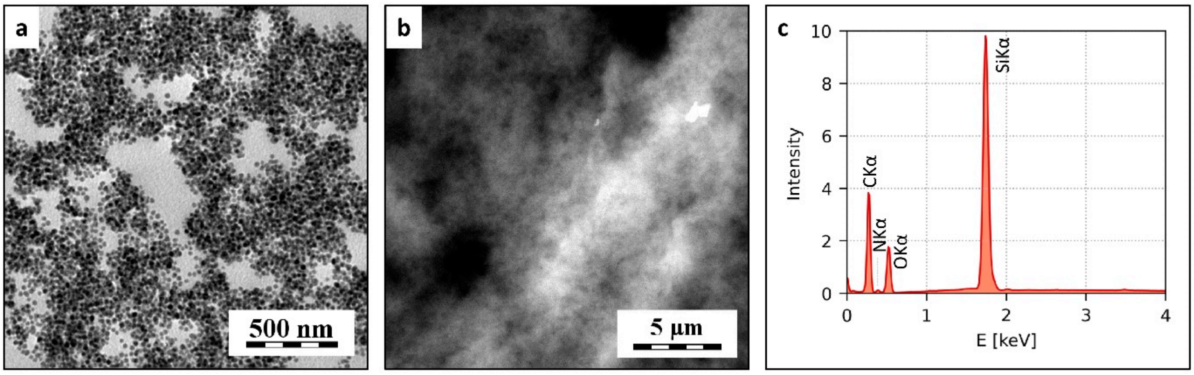

Figure S1. *Morphology of PUU/nanosilica composite with 30% of silica: (a) TEM/BF micrographs, (b) SEM/BSE micrographs, and (c) SEM/EDX spectra.*

4 Ammonia adsorbed on the silica nanospheres

The presence of ammonia on nanofiller surface (see **SI-Scheme S3**) plays an important role in the mechanical properties. Its adsorption is necessary for stabilizing the aqueous dispersion of this nanofiller, which is then indefinitely storable even at a weight concentration of 30%: The positive charge on the nanospheres' surface prevents them from growing-together via the condensation of Si–OH groups.

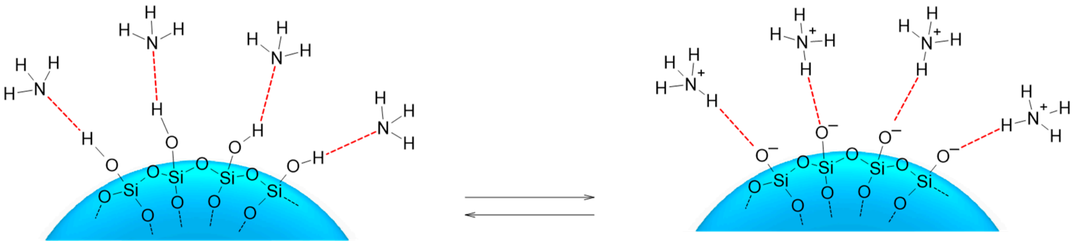

Scheme S3. *Adsorption of ammonia on the surface of silica nano-spheres: oscillation between hydrogen bonding (Si–OH to ammonia) and ammonium polysilicate formation.*

The ammonia groups on the surface of nanoSiO_2 can make the same hydrogen bonding like the unprotected Si–OH groups, as it was depicted in **Scheme 2** the main Manuscript file, but with ammonia, the hydrogen bonds to the hard or soft blocks of the PU matrix will be weaker. This is due to the protons of ammonia being less activated (acidic) than those of Si–OH. Moreover, the ammonia molecules are not strongly bonded to fixed positions, but can 'slide' via desorption/adsorption. The above-mentioned aspects explain, why the silica nanofiller raises the rubbery modulus by only one order of magnitude (even at 30 wt.%), after being embedded into a strongly hydrogen-bonding polyurethane matrix, in spite of silica being strongly hydrogen-bonding, and in spite of its tremendously high specific surface. A distinct **weakening of interfacial hydrogen bonding by ammonia** adsorbed on nano-silica was noted in the literature for polyurethane–SiO_2 nanocomposites **[30]**, and also was observed by the authors of this work in the case of polyacrylamide nanocomposites reinforced with ammonia-stabilized (commercial) vs. non-stabilized (in-situ-formed) nano-silica **[50]**. Nevertheless, the stabilized nano-silica still is an attractive nanofiller, also for model studies like this one, because of its nearly perfect spherical shape and uniform small size.

References (same numbers like in main manuscript file):

[30] Heck C A, dos Santos J H Z, Wolf C R: Waterborne polyurethane: the effect of the addition or in situ formation of silica on mechanical properties and adhesion, *International Journal of Adhesion and Adhesives 58* **(2015)** 13–20. https://doi.org/10.1016/j.ijadhadh.2014.12.006 .

[50] Strachota B, Šlouf M, Matějka L: Tremendous reinforcing, pore-stabilizing and response-accelerating effect of in situ generated nanosilica in thermoresponsive poly(N-isopropylacrylamide) cryogels, *Polymer International 66* **(2017)** 1510–1521. https://doi.org/10.1002/pi.5406 .

5 Interface interactions (FTIR)

The studied PUU/silica nanocomposites were analysed by means of FTIR in the previous work **[1]** (cited as **[1]** also in the main manuscript), in order to elucidate the interactions on the matrix–nanofiller interface, although the FTIR discussion was not very detailed. A more dedicated discussion of the spectra from **[1]** is provided here:

The increasing concentration of the silica nanofiller generally led to a proportional decrease of the signals of the organic PUU matrix.

Besides simple concentration effects, a significant intensity change was observed for the peak at 1240-1246 cm^{-1}, assigned to asymmetrical C-O-C stretching in both urethane and carbonate groups, which faded much more than proportionally with increasing filler content.

An analogous diminishing trend like above also was observed for the peaks at 1626 and 1580 cm^{-1}, which were assigned to hydrogen-bonded C=O groups of urethane and urea, respectively **[1]**.

The signals in the region of N–H and O–H stretching (3350 cm^{-1}), as well as the ones in the C–H stretching region (near 2855 and from 2911 to 2947 cm^{-1}) became les structured with increasing filler content **[1]**.

The free carbonyl groups of urethane units (hard segments of the PUU matrix) and of the carbonate units (soft segments of matrix) both absorb at 1739 cm^{-1}, and their single peak was dominated by the signal of not-hydrogen-bonded carbonate (which is present in large excess, due to the dominant mass fraction of the elastic polycarbonatediol chains in the matrix), so that it did not disproportionally decrease with increasing silica content **[1]**.

The above results hence suggest changes in the hydrogen bonding of the urethane and possibly also of the carbonate units, as well as a less ordered arrangement of the matrix.

Reference (same number like in main manuscript file)**:**

[1] Spirkova M, Pavlicevic J, Costumbre Y A, Hodan J, Krejcikova S, Brozova L: Novel waterborne poly(urethane-urea)/silica nanocomposites. *Polymer Composites 41* **(2020)** 4031–4042. https://doi.org/10.1002/pc.25690 .

6 DMTA: Thermo-mechanical properties

6.1 Comparison of Storage and Complex modulus

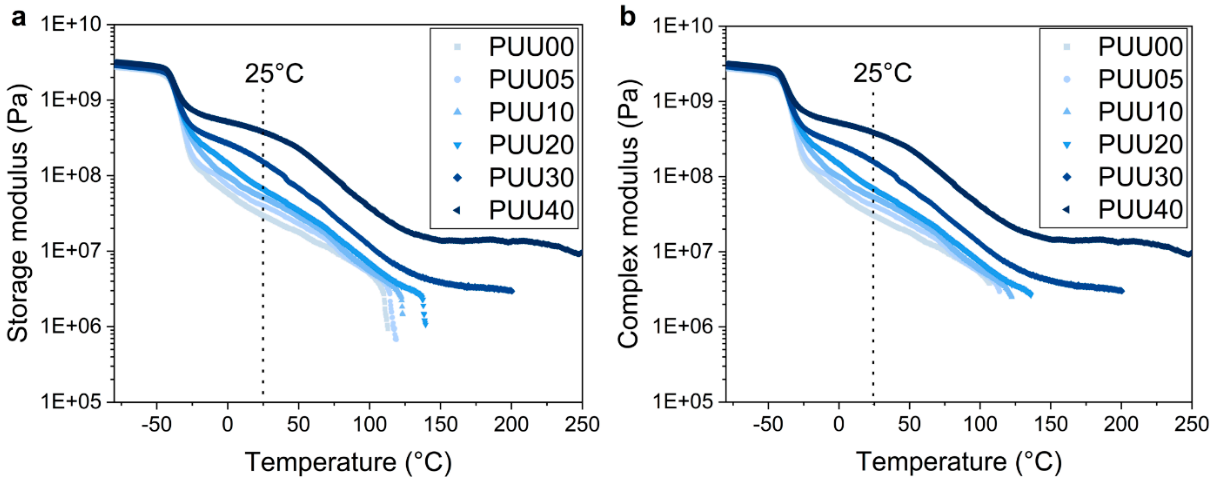

Figure S2. *DMTA curves for PUU/nanosilica composites. The curves show (a) storage modulus, and (b) absolute value of complex modulus for all investigated filler concentrations.*

In **SI-Figure S2b**, the temperature-dependent complex moduli G* (= G’ + *i* G”) are plotted: they show practically identical trends like G’ (**SI-Figure S2a**), because G’ >> G”.

6.2 Description of temperature-dependent trends of Storage- (G') and Loss modulus (G"), as well as of the loss factor tan(delta)

The temperature-dependent graphs of storage- (G'), of loss modulus (G"), as well as of the loss factor tan(delta), which are shown in **Main-Manuscript-Figure 2**, display several characteristic temperature regions: the glassy region (from –80 °C to ca. –45 °C), the region of the glass transition (from ca. –45 °C to ca. –15 °C), the lower-temperature rubbery region (affected by hydrogen bridging and reaching from ca. –15 °C to ca. +100 °C), the region of abrupt melting – only in the samples PUU00 till PUU20 (with increasing filler loading the melting range shifts from 108 °C to 145 °C), the high-temperature rubbery region – only in the samples PUU30 and PUU40: (from ca. 140 °C to ca. 200 °C), and, finally, the region of thermal degradation (above ca. 200 °C; it was investigated only in the samples PUU30 and PUU40) The temperature-dependent trends of the DMTA magnitudes are discussed below.

Glassy region:
No difference between the samples.

near T_g: Glass transition region:
Near –30 °C a glass transition is observed, which manifests itself as a **steep step in the storage modulus G'** (see **Main-Manuscript-Figure 2a**: step position between –36 °C for the sample with 40 wt.% silica, and –32 °C for the neat matrix). This same transition also generates a distinct **peak in the loss modulus G"** (see **Main-Manuscript-Figure 2b**: peak maximum at -37 °C for all samples), and equally in **tan(delta)** (see **Main-Manuscript-Figure 2c**: peak maximum between –35 °C for the sample with 40 wt.% silica and –28 °C for the neat matrix). The physico-chemical meaning **[45]** of the glass transition is, that the conformational movement of the elastic chains (originating from the polycarbonate component) in the PU matrix is enabled at temperatures above the glass transition temperature (T_g), thus making possible rubber-like elastic deformations.

Rubbery region affected by hydrogen bridging:
In the range from –15 °C to +100 °C, an imperfect rubbery region extends, in which the storage modulus **G'** (see **Main-Manuscript-Figure 2a**) steadily decreases with rising temperature, from very high values (as for a rubber) down to 'normal rubbery values' (expected: units of MPa, according to p. 91 in **[45]**). On the example of the neat PU matrix, G' decreases from 100 MPa down to 4 MPa in this region. In case of typical rubbers, G' should moderately increase with temperature in the rubbery region ('plateau'), and its value should be in units of MPa **[45]**. The **G' = f(T) curves** in the rubbery region **shift up with increasing filler loading** in the samples, especially at 30 and at 40 wt.% of nano-SiO_2 (14.6 and 21.1 v/v%, respectively). With 40 wt.% of SiO_2, the storage modulus is 1 order higher than in the neat matrix at the same

temperature value. This **reinforcement** is much higher than could be expected from the hydrodynamic effect (see **[48]**) caused by a rigid filler (ca. a 1.2-fold increase would be expected), and it must be mostly attributed to matrix–nanofiller interactions, namely to mutual physical **crosslinking by hydrogen bonds,** as both the PUU matrix and the nanosilica filler are materials able of efficient hydrogen bonding.

The **strongly decreasing course of G' = f(T)** suggests the **thermally-induced disconnection of** reversible crosslinks (see **[45]**), namely of **hydrogen bonds,** whose strength has a wide distribution. Their gradually progressing reversible dissociation with increasing temperature is further documented by peaks in tan(delta) and G" (see **Main-Manuscript-Figure 2b,c**) which are discussed below. At the end of the lower-temperature part of the rubbery region, the curves of the storage modulus G' approach (but do not achieve) a horizontal course. Except in case of the highest-filled samples **PUU30 and PUU40**, all the other nanocomposites abruptly melt at the end of the mentioned temperature region, while the highest-filled ones display a nearly ideal **'second' rubbery plateau in the temperature region above 100 °C**, until thermal degradation above 200 °C. This suggests that in the highest-filled samples, a fraction of physical crosslinks is thermally irreversible (namely the H-bonding between hard segments of the matrix and silica surface, as will be discussed).

If the **trends of loss modulus G"** (see **Main-Manuscript-Figure 2b**) **and of tan(delta)** (see **Main-Manuscript-Figure 2c**) **in the lower-temperature rubbery region** (ca. from –15 °C to +110 °C) are considered, it can be observed that the tan(delta) = f(T) (**Main-Manuscript-Figure 2c**) curves display a large peak centred near the end of this region (near 100 °C). This large peak becomes higher and broader with increasing filler loading, especially in the highly filled samples PUU30 and PUU40. Additionally, in the case of the less filled samples PUU00 till PUU20, a new small tan(delta) peak near +12 °C is clearly observed. In case of PUU30 and PUU40, instead of the mentioned small peak, there is just a shoulder which already finds itself on the slope of the large peak near 100 °C, and this shoulder is shifted from 12 °C to 50 °C (PUU30), or to 70 °C (PUU40). In case of the curves of G" = f(T), (**Main-Manuscript-Figure 2b**) peaks that would be analogous to the ones observed for tan(delta) are practically invisible, except the lower-temperature-peak in PUU30 and PUU40 (which was not prominent in the tan(delta) curves). In the very latter case (PUU40), however, the G" peak near 70 °C is prominent and very broad. Except for the mentioned G" peaks in PUU30 and PUU40, the loss modulus G" practically steadily decreases with T in the whole rubbery region in all the samples. The trends of tan(delta) and G" distinctly differ from the ones observed for simple linear semicrystalline polymers (see example in **SI-Figure S3a** where one of the specimens prepared in **[49]** is analysed), or for simple elastomeric networks (see example in **SI-Figure S3b:** a specimen synthesized in the study **[2]**). In case of the meltable semicrystalline polymer in **SI-Figure S3a**, a somewhat imperfect plateau in tan(delta) is observed after the T_g peak, in the rubbery region between T_g and the melting point. At the melting point, a high upward step in tan(delta) occurs, followed by a plateau at high tan(delta) values (melt region). In the typical elastomeric network (**SI-Figure S3b**), the trend is simpler: a perfect plateau of the tan(delta) curve is observed above T_g.

Assignment of the tan(delta) peaks to the disconnection of different types of hydrogen bonds: In contrast to the above-discussed references, the studied nanocomposites do not display even an imperfect plateau in tan(delta) in the rubbery region, but peaks instead. The small peak (or shoulder) at lower temperatures can be assigned to the dissociation of weak hydrogen bonds. The large tan(delta) peak near 100 °C can be assigned to the dissociation of strong H-bonds between hard segments. The up-shift of the small peak which becomes a shoulder on the 100 °C peak can be seen as the result of overall matrix immobilization at high filler contents.

The meltable samples (from PUU00 to PUU20) additionally do not display the high plateau of tan(delta) after the melting point, like the linear polymer from **SI-Figure S3a**, but oppositely a decrease of tan(delta) above T_m. The mentioned differences in the course of tan(delta) = f(T), suggest that energy-consuming processes occur in the rubbery region in the nanocomposites (indicated by the tan(delta) peaks). In connection with the above-discussed trend of G', it can be postulated that the energy-consuming process is the disconnection of sufficiently weak hydrogen bonds (matrix–matrix and matrix–filler). The decrease of tan(delta) in the molten samples suggests the persistence of significant elasticity in the molten state, presumably due to residual (and dynamic) hydrogen bonding.

Melting region:

Between 108 and 145 °C (depending on filler content) abrupt melting is observed for all the samples except those with 30 and 40 wt.% of silica – the latter do not melt altogether. The melting manifests itself as a very steep step in G', and as a broad but prominent peak in tan(delta), which marks the end of the rubbery region. Interestingly, in the curves of the loss modulus G" (**Main-Manuscript-Figure 2b**), there is no peak (nor at least a step upwards) at this transition, but a moderately steep step down (less abrupt than in G'), except for specimens with filler loading of 30 or 40 wt.% (the mentioned infusible samples), where no melting step in G" is observed, but a continuation of the steady G" decrease, until the region of thermal degradation above 200 °C. The combination of the trends of G' and G" gives interesting hints concerning molecular dynamics and hydrogen bonding, which will be discussed further below.

Thermal degradation region:

Above 200 °C, thermal degradation sets on, and becomes marked near 250 °C. This was observed visually, and in case of PUU40, the degradation can be noted in **Main-Manuscript-Figure 2** as a simultaneous drop in all mechanical magnitudes (G', G", as well as tan(delta)).

References (same numbers like in main manuscript file):

[2] Slouf M, Strachota B, Strachota A, Gajdosova V, Bertschova V, Nohava J: Macro-, micro- and nanomechanical characterization of crosslinked polymers with very broad range of mechanical properties. *Polymers 12* **(2020)** 2951/1–2951/26. https://doi.org/10.3390/polym12122951 .

[45] Mezger T G: *The Rheology Handbook For users of rotational and oscillatory rheometers,* 4th ed.; Vincentz Network: Hanover, Germany, **2014**, ISBN13: 978-3-86630-650-9.

[48] Heinrich G, Kluppel M, Vilgis T A: Reinforcement of elastomers. *Current Opinion in Solid State and Materials Science 6* **(2002)** 195–203. https://doi.org/10.1016/S1359-0286(02)00030-X .

[49] Gajdosova V, Strachota B, Strachota A, Michalkova D, Krejcikova S, Fulin P, Nyc O, Brinek A, Zemek M, Slouf M: Biodegradable Thermoplastic Starch/Polycaprolactone Blends with Co-Continuous Morphology Suitable for Local Release of Antibiotics. *Materials 15* **(2022)** 1101. https://doi.org/10.3390/ma15031101 .

6.3 Reference materials: DMTA

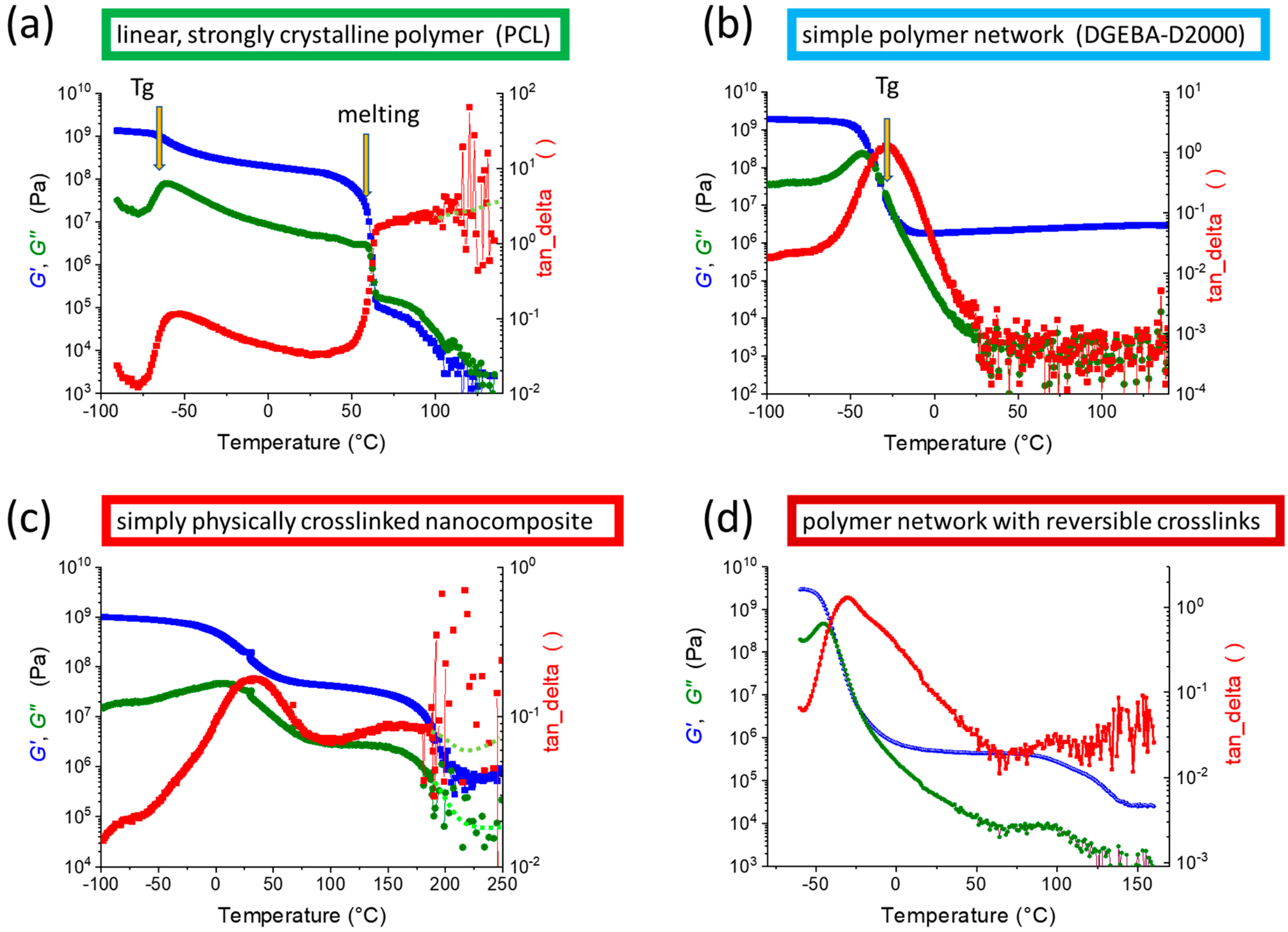

Figure S3. *Examples of DMTA profiles of typical polymeric, simple polymer nanocomposite, as well as reversibly crosslinking (polymeric) materials, the specimens of which were prepared in the course of authors' previous research: (a) linear semicrystalline polymer: polycaprolactone with a high degree of crystallinity and with Mn = 80 000 g/mol from* **[49]**; *(b) simple polymer network which is rubbery at room temperature: the epoxy system diglycidyl ether of bisphenol A (DGEBA) – polypropyleneoxide-α,ω-diamine ('Jeffamine D2000') from* **[2]**; *(c) simple, partly physically crosslinked polymer network, in which the inorganic POSS nano-building blocks assemble to nano-aggregates and thus act as reversible crosslinker: system based on DGEBA-co-possDGEBAoligomer)–D2000 from* **[55]**; *(d) polymer network in which a part of crosslinks is chemically reversible: system {network DGEBA–D2000 interpenetrated by network polypropyleneoxideBisMaleimide–tetrakisFurfurylD2000} from* **[56]**.

References (same numbers like in main manuscript file):

[2] Slouf M, Strachota B, Strachota A, Gajdosova V, Bertschova V, Nohava J: Macro-, micro- and nanomechanical characterization of crosslinked polymers with very broad range of mechanical properties. *Polymers 12* **(2020)** 2951/1–2951/26. https://doi.org/10.3390/polym12122951 .

[49] Gajdosova V, Strachota B, Strachota A, Michalkova D, Krejcikova S, Fulin P, Nyc O, Brinek A, Zemek M, Slouf M: Biodegradable Thermoplastic Starch/Polycaprolactone Blends with Co-Continuous Morphology Suitable for Local Release of Antibiotics. *Materials 15* **(2022)** 1101. https://doi.org/10.3390/ma15031101 .

[55] Strachota A, Kroutilová I, Kovářová J, Matějka L: Epoxy Networks Reinforced with Polyhedral Oligomeric Silsesquioxanes (POSS). Thermomechanical Properties. *Macromolecules 37* **(2004)** 9457–9464. https://doi.org/10.1021/ma048448y .

[56] Strachota B, Hodan J, Dybal J, Matějka L: Self-Healing Epoxy and Reversible Diels-Alder Based Interpenetrating Networks. *Macromolecular Materials and Engineering 306* **(2021)** 2000474. https://doi.org/10.1002/mame.202000474 .

7 Discussion of thermo-mechanical properties (DMTA): effects of structure and morphology

The **Main-Manuscript-Figure 8** evaluates the trends (dependence on filler loading) and correlations of the macro-mechanical properties which were analyzed by DMTA (see **Main-Manuscript-Figure 2**). These trends and correlations are discussed here in detail in view of matrix–matrix and matrix–nanofiller interactions (illustrated in **Main-Manuscript-Scheme 1** and **2**).

In **Main-Manuscript-Figure 8a,b** it can be seen that (at 25 °C) **G' and G" display practically identical trends if the filler amount is varied**, but G" always is nearly 1 order smaller. Observed in the linear scale, both G' and G" moderately and nearly linearly increase with filler content between 0 and 20 wt.% of silica. Above 20 wt.%, both moduli increase more dramatically, G' (elasticity-related) more so, than G" (energy-dissipation-related). This small difference appears to have its origin in the samples' morphology (see **Main-Manuscript-Figure 1**): At 40 wt.% (21 v/v%) of nano-silica, the pattern of the rigid filler is 'practically percolating'. This further raises elasticity, in addition to increased hydrogen bonding (due to higher interfacial area). A similar additional 'percolation effect' is missing for G", at least at the small deformations endured in DMTA tests, so that G" increases less steeply between 30 and 40% of SiO_2 in **Main-Manuscript-Figure 8a,b**.

An **unusual feature is, that G" is relatively high in comparison to G'** at T = 25 °C, and also **in the whole rubbery region** (see **Main-Manuscript-Figure 2** and **8**). This is true not only for the nanocomposites, but even for the neat matrix (see its DMTA profile in **Main-Manuscript-Figure 2**). Characteristic reference materials (see **SI-Figure S3**) can be compared with the here studied PUU series. The 'reference specimens' include a linear semi-crystalline polymer, related to materials studied in **[49]**, a simple elastomeric polymer network which was investigated in **[2]**, a nanocomposite displaying physical crosslinking via nano-aggregation of pendant groups which was prepared in **[56]**, and a network with reversible covalent bonds which was synthesized in **[57]**. It can be seen, that in the simple elastomeric network in its rubbery region (**SI-Figure S3b**), G" is smaller than G' by three orders of magnitude. In the less perfectly crosslinked linear semicrystalline polymer, G'/G" = ca. 100 (tan(delta) = 0.01), similarly like in the partly reversible covalent network (both in the rubbery region). The smallest G'/G" ratio (highest tan(delta)) in the rubbery region is found in case of the nanocomposite with physical crosslinks generated by pendant unit aggregation. The latter nanocomposite, as well as the network with reversible covalent crosslinks, both display a similar course of tan(delta) = f(T) in their rubbery regions, like the here studied PUU matrix and its nanocomposites (compare **SI-Figure S3c,d** and **Main-Manuscript-Figure 2**). In both the latter reference materials, it can be expected that some of their **reversible crosslinks dynamically disconnect and re-connect** (due to local mechanical strains) in the whole rubbery region of temperatures.

In view of the theoretical discussion of matrix–matrix and matrix–filler interactions (see section 4.1. of the Main Manuscript), as well as of the resulting assignment of transitions in DMTA curves in **Main-Manuscript-Figure 2**, it can be suggested, that the dominant **special effect** in the rubbery region (hence also at T = 25 °C) of both the neat PUU matrix, as well as of its nanocomposites, is the hydrogen bonding to the 'soft' and flexible (elastic) polycarbonate (PCD) blocks (see **Main-Manuscript-Scheme 1c1,c2** and **Main-Manuscript-Scheme 2a** and **[58]**). In case of the neat matrix, only the urethane-unit-rich hard blocks can connect by **moderately strong hydrogen bonds to the soft PCD blocks** (**Main-Manuscript-Scheme 1c1,c2**), while in the nanocomposites, also the silica nanospheres can be expected to undergo analogous weak hydrogen bonding (**Main-Manuscript-Scheme 2a**). The effect of the PCD–nanoSiO_2 hydrogen bonding raises the rubbery modulus (and hence the elasticity) by 1 order of magnitude at 40 wt.% (21 v/v%) of SiO_2, which by far exceeds the theoretical (see **[48]**) hydrodynamic effect of the filler (as noted further above).
On the other hand, the weak nature of the hydrogen bonds to PCD was assigned to be responsible for the temperature-induced distinct decrease of the rubbery modulus in **Main-Manuscript-Figure 2** (as discussed above). According to the authors' experience from previous work **[59]**, weak thermo-reversible physical crosslinks typically also can be reversibly disconnected mechanically, even at relatively moderate deformations. This effect then leads to fairly high tan(delta) values in mechanical tests **[59]**, because at any given temperature in the rubbery region of such elastomers, a fraction of mechanically labile physical crosslinks exists. Such labile crosslinks make possible an energy-absorption mechanism: Upon deformation larger than very small, some of them are disconnected by the mechanical force (energy absorption), and subsequently they reconnect (exothermically under heat release) at spatially shifted positions, so that in total, mechanical energy is converted to heat. Hence, the relatively weak PCD–nanoSiO_2 hydrogen bonding raises not only G', but also the loss modulus G'' (related to energy dissipation).

Relative tendency to energy dissipation vs. energy storage expressed as tan(delta): complex trends different from G" (compare **Main-Manuscript-Figure 8b** vs. **8c**)**:**
In thermo-mechanical analysis, the loss modulus G" quantifies the tendency of solids to dissipate energy upon deformation, while the loss factor tan(delta) = G"/G' expresses the relative tendency to energy dissipation (vs. elastic energy storage).
It can be observed that tan(delta) (in **Main-Manuscript-Figure 8c**) at first modestly decreases with filler content, if going from 0 to 5 wt.% of silica: This means an increase of the relative elastic character. The effect can be attributed to the additional crosslinking by hydrogen bonds nanofiller–matrix (see **Main-Manuscript-Scheme 2a,b**), which still is not disconnected at the small deformations in DMTA, and possibly also to the 'insular' distribution of the nanofiller (see **Main-Manuscript-Figure 1a**) which might support elasticity at moderately large deformations (as will be discussed also in Main Manuscript section 4.2.5.).
Next, between 5 and 20 wt.% of silica, the relative tendency to energy dissipation (tan(delta)) modestly increases. The weak and labile hydrogen bonding between PCD chains and the more evenly distributed nano-silica (**Main-Manuscript-Scheme 2a**) becomes more often

mechanically dissociated, because the relatively large filler-free soft and elastic (at low deformations) domains disappear.

Finally, between 20 and 40 wt.%, and especially between 30 and 40 wt.% of silica, the relative relative tendency to energy dissipation (tan(delta)) decreases again, which is likely the result of the already mentioned filler percolation (see **Main-Manuscript-Figure 1** further above).

References (same numbers like in main manuscript file)**:**

[1] Spirkova M, Pavlicevic J, Costumbre Y A, Hodan J, Krejcikova S, Brozova L: Novel waterborne poly(urethane-urea)/silica nanocomposites. *Polymer Composites 41* **(2020)** 4031–4042. https://doi.org/10.1002/pc.25690 .

[2] Slouf M, Strachota B, Strachota A, Gajdosova V, Bertschova V, Nohava J: Macro-, micro- and nanomechanical characterization of crosslinked polymers with very broad range of mechanical properties. *Polymers 12* **(2020)** 2951/1–2951/26. https://doi.org/10.3390/polym12122951 .

[45] Mezger T G: *The Rheology Handbook For users of rotational and oscillatory rheometers,* 4th ed.; Vincentz Network: Hanover, Germany, **2014**, ISBN13: 978-3-86630-650-9.

[48] Heinrich G, Kluppel M, Vilgis T A: Reinforcement of elastomers. *Current Opinion in Solid State and Materials Science 6* **(2002)** 195–203. https://doi.org/10.1016/S1359-0286(02)00030-X .

[49] Gajdosova V, Strachota B, Strachota A, Michalkova D, Krejcikova S, Fulin P, Nyc O, Brinek A, Zemek M, Slouf M: Biodegradable Thermoplastic Starch/Polycaprolactone Blends with Co-Continuous Morphology Suitable for Local Release of Antibiotics. *Materials 15* **(2022)** 1101. https://doi.org/10.3390/ma15031101 .

[56] Strachota B, Hodan J, Dybal J, Matějka L: Self-Healing Epoxy and Reversible Diels-Alder Based Interpenetrating Networks. *Macromolecular Materials and Engineering 306* **(2021)** 2000474. https://doi.org/10.1002/mame.202000474 .

[57] Strachota B, Hodan J, Dybal J, Matějka L: Self-Healing Epoxy and Reversible Diels-Alder Based Interpenetrating Networks. Macromolecular Materials and Engineering 306 (2021) 2000474. https://doi.org/10.1002/mame.202000474 .

[58] Špírková M, Pavličević J, Strachota A, Poreba R, Bera O, Kaprálková L, Baldrian J, Šlouf M, Lazić N, Budinsky-Simendic J: Novel polycarbonate-based polyurethane elastomers: composition-property relationship. European Polymer Journal 47 (2011) 959–972. https://doi.org/10.1016/j.eurpolymj.2011.01.001 .

[59] Horodecka S, Strachota A, Mossety-Leszczak B, Kisiel M, Strachota B, Šlouf M: Low-Temperature-Meltable Elastomers Based on Linear Polydimethylsiloxane Chains Alpha, Omega-Terminated with Mesogenic Groups as Physical Crosslinker: A Passive Smart Material with Potential as Viscoelastic Coupling. Part II—Viscoelastic and Rheological Properties. Polymers 12 (2020) 2840. https://doi.org/10.3390/polym12122840 .

8 Elastic indentation properties: Discussion

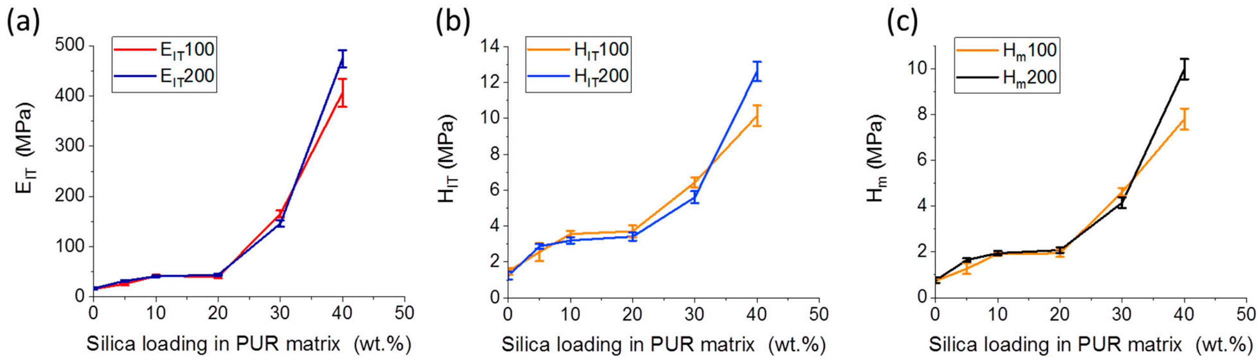

Figure S4. *Comparison of trends of elasticity-related magnitudes measured by microindentation, as function of nanofiller loading, all measured at T = const. = 25 °C: (a) indentation modulus* E_{IT}, *(b) indentation hardness* H_{IT}, *(c) Martens hardness* H_M.

9 Energy-dissipation-related indentation properties: Discussion

At **0 to 5(10 wt.%) of SiO2:** In contrast to tan(delta) and similarly like G”, the indentation creep C_{IT} first increases with silica loading. This can be attributed to the further-above-discussed (section 4.2.3.) suppression of larger crystallite-like lamellae by nanosilica–matrix interactions (as observed in **[1]**). In case of C_{IT}, relatively large local deformations occur in the material in the close neighborhood of the indentation tool. The suppression of the formation of the large and elastic lamellae seems to outweigh the effect of the small amount of newly formed but weak crosslinking matrix–silica. The weak crosslinks can yield at higher deformations (cold flow, which contributes to energy-dissipating character’).

5(10%) to 20 wt.% of SiO2: The C_{IT} increase becomes weaker between 5 and 10 wt.% of nano-silica loading, and, next, C_{IT} decreases between 10 and 20% (this means more elasticity). This behavior can be correlated with the presence of numerous prominent and spatially separated silica-rich domains (see morphology in **Main-Manuscript-Figure 1**, as well as **Main-Manuscript-Scheme 3**). These ‘filler islands’ could act as a physical crosslinker in the matrix on the meso-scale, thus connecting the polymer chains from the filler-deficient domains to an infinite elastic network. Due to the relatively large size of the highly elastic filler-deficient domains (**Main-Manuscript-Figure 1**), the network might be better able to purely elastically resist the large local deformations which occur during the indentation test. G” and tan(delta) grow in this region, most likely due to an increasing fraction of mechanically easily dissociable (even at low deformations) H-bonds (see interactions in **Main-Manuscript-Scheme 2a**).

20 to 40 wt.% of SiO_2: If going from 20 to 40 wt.% of silica, C_{IT} increases again with filler content, and even distinctly, especially between 20 and 30%. The loss modulus G” also increases in this filler-loading range. The trend correlates with the gradual diminishment of the filler-free domains (see **Figure 1d,h** further above). The trend of rising energy dissipation hence could reflect the increased amount of weak and easily mechanically disconnecting hydrogen bonding nanoSiO_2–PCD (shown in **Scheme 2a**), as well as the disappearance of larger filler-deficient elastic and extensible domains (shown in **Scheme 3**). Even moderate deformations hence necessitate the disruption of hydrogen bonding. The trend of tan(delta) = f(filler concentration) is opposite to C_{IT} and G”, if going from 20 to 40 wt.% of SiO_2: This means an increase in relative elasticity, and can be attributed to the practically percolating distribution of the filler (the latter supports elasticity, as discussed further above in the section 4.2.3.), as well as to the fact that the deformations endured in DMTA are small (note: G” increase signalizes the increase of energy dissipation on the absolute scale; G’ grows even more strongly, hence that tan(delta) = G”/G’ decreases at the same time).

<u>**Reference** (same number like in main manuscript file)**:**</u>

[1] Spirkova M, Pavlicevic J, Costumbre Y A, Hodan J, Krejcikova S, Brozova L: Novel waterborne poly(urethane-urea)/silica nanocomposites. *Polymer Composites 41* **(2020)** 4031–4042. https://doi.org/10.1002/pc.25690 .

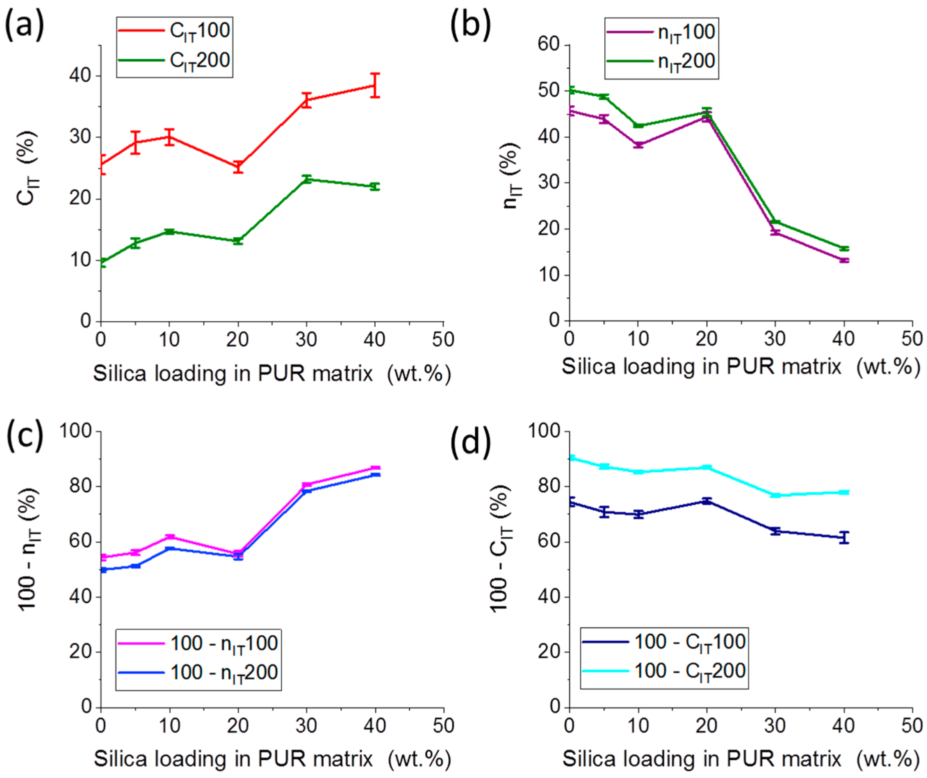

Figure S5. *Comparison of trends and correlations of energy-dissipation-related magnitudes measured by microindentation, as function of nanofiller loading, all measured at T = const. = 25 °C: (a) indentation creep* C_{IT}*, (b) indentation work* n_{IT}*, (c) 100 –* n_{IT}*; and (d) 100 –* C_{IT}*.*
